# Increasing Olanzapine Prescribing for Patients Undergoing Highly Emetogenic Chemotherapy

**DOI:** 10.1001/jamanetworkopen.2025.10392

**Published:** 2025-05-21

**Authors:** Ashley M. Bowen, Aimee Cloud, Suzanne Fadly, Ryan Gennette, Zachary Hector-Word, Joann Hirth, Yelena Kier, Diana Kostoff, Philip Kuriakose, Binu Malhotra, Houman Nourkeyhani, Jatin Rana, Keli DeVries, Emily Mackler, Shawn Winsted, Eric Voisine, Jennifer J. Griggs

**Affiliations:** 1 Michigan Oncology Quality Consortium, Ann Arbor; 2 Munson Healthcare, Traverse City, Michigan; 3 Karmanos Cancer Institute at McLaren Greater Lansing, Michigan; 4 Henry Ford Health, Jackson, Michigan; 5 Henry Ford Health, Detroit, Michigan; 6 Covenant Healthcare, Saginaw, Michigan; 7 The Cancer and Hematology Centers, Grand Rapids, Michigan; 8 University of Michigan Department of Medicine (Hematology/Oncology) and Policy, Ann Arbor

## Abstract

**Importance:**

Olanzapine as part of a 4-drug antiemetic regimen is highly effective at preventing nausea and vomiting in patients receiving highly emetogenic chemotherapy (HEC). National prescribing rates of olanzapine in eligible patients have, however, remained persistently low.

**Objective:**

To describe efforts to increase the guideline-concordant use of olanzapine in patients receiving HEC in a statewide oncology collaborative.

**Design, Setting, and Participants:**

The Michigan Oncology Quality Consortium (MOQC) is a collaborative of Michigan-based oncology practices whose mission is to advance the care of patients with cancer and their caregivers through comprehensive, patient- and practice-led quality improvement initiatives. Participants were patients receiving HEC at 38 MOQC member practices during an initiative from 2019 to 2023 to improve guideline-concordant inclusion of olanzapine as part of a 4-drug antiemetic regimen in HEC prophylaxis.

**Interventions:**

Interventions included performance audit and feedback to practices along with peer comparison, learning collaboratives of practices, education by experts at collaborative meetings, creation of patient-facing materials, and the addition of value-based reimbursement beginning in 2021. Measure performance was assessed using the American Society of Clinical Oncology’s Quality Oncology Practice Initiative.

**Main Outcomes and Measures:**

Proportion of patients receiving olanzapine for the first cycle of HEC as part of a 4-drug antiemetic regimen for chemotherapy-induced nausea and vomiting.

**Results:**

Between 2019 and 2024, data were manually collected from the medical records of 8662 patients treated with HEC at 38 medical oncology practices at 71 sites across the state. Individual patient-level demographic data are not available from the QOPI database. For patients from years 2021 through 2024, the median (IQR) age was 62 (52-69) years; 4434 were female (65.5%); 814 were Black or African American (12.0%), 121 were Hispanic or Latino (1.8%), and 5385 were White (79.7%). The use of olanzapine as part of a 4-drug antiemetic increased from 7.2% in 2019 to 63.4% in 2024 (χ^2^_1_ = 553.61; *P* < .001).

**Conclusions and Relevance:**

In this quality improvement study of olanzapine prescribing in eligible patients, MOQC deployed quality improvement methods to increase the prescribing of olanzapine as part of a 4-drug regimen in patients receiving HEC, well above both the baseline and national levels of prescribing. This work suggests that efforts to improve patient care can be effective across diverse practice types, locations, and sizes in a statewide collaborative. Ongoing efforts include supporting change in those practices with low rates of prescribing and demonstrating the importance of this work for patient outcomes.

## Key Points

**Question:**

Can guideline-concordant olanzapine prescribing for patients receiving highly emetogenic chemotherapy be boosted through a quality improvement initiative in a statewide oncology collaborative?

**Findings:**

In this quality improvement study of 8662 patient records, the initiative, including audit and feedback, education, learning collaboratives, patient-facing materials, and value-based reimbursement, was associated with an increase in olanzapine prescribing from 7.2% to 63.4% over 5 years, surpassing the national average and the collaborative’s target.

**Meaning:**

These findings suggest statewide strategies were significantly associated with increased guideline-concordant olanzapine use, potentially improving patient quality of life and reducing costs.

## Teaser

This quality improvement study evaluates efforts to increase the guideline-concordant use of olanzapine in patients receiving highly emetogenic chemotherapy in a statewide oncology collaborative.

## Introduction

Chemotherapy-induced nausea and vomiting (CINV) is an important adverse effect of cancer treatment that negatively affects quality of life^1,2,3^ and increases resource utilization and costs of care.^4,5^ Olanzapine is highly effective in preventing CINV, including delayed nausea and vomiting, as part of a 4-drug regimen along with a corticosteroid, a neurokinin-1 receptor antagonist (NK1RA), and a selective serotonin receptor antagonist (5HT3RA)^6,7,8,9^ in patients receiving highly emetic chemotherapy (HEC). Olanzapine, an atypical antipsychotic with a high affinity for multiple dopamine and serotonin receptors, is particularly effective in reducing the risk of delayed nausea and vomiting.^6,7,8,9^ Based on multiple randomized trials demonstrating the safety and efficacy of olanzapine in patients receiving HEC, the clinical practice guidelines of the American Society of Clinical Oncology (ASCO),^10^ the National Comprehensive Cancer Network,^11^ and the Multinational Association of Supportive Care in Cancer/European Society of Medical Oncology collaborative guidelines^12^ all recommend the use of olanzapine on the day of chemotherapy and for the following 3 days for these patients beginning with the first cycle of HEC.

This project was selected based on national olanzapine prescribing data and on discussions at our collaborative meetings that indicated there was substantial underuse of olanzapine. The proportion of people receiving HEC who are prescribed olanzapine nationally has remained persistently low despite the drug’s inclusion in the guidelines as well as its efficacy, low rates of toxic effects, and low cost. In a commercially insured patient sample, only 14% of eligible patients received olanzapine in 2021.^13^ At the time the initiative began in 2019, olanzapine was prescribed in only 7% of patients receiving HEC in the Michigan Oncology Quality Consortium (MOQC) based on medical record audit.

The purpose of this initiative was to increase prescribing of olanzapine as part of a 4-drug antiemetic regimen in patients receiving their first cycle of HEC in the diverse practices that are part of the MOQC using the Knowledge-to-Action framework^14^ for quality improvement. The Knowledge-to-Action approach to quality improvement allows practices to adapt resources to their local environment and needs and to conduct their own quality improvement interventions.

## Methods

This study followed the Standards for Quality Improvement Reporting Excellence (SQUIRE) 2.0 reporting guideline.^15^ This study was deemed exempt by the University of Michigan institutional review board (IRB). Patient consent was waived by the IRB because data were deidentified. All MOQC practices have participation and data use agreements with MOQC.

### Context

MOQC is a collaborative of oncology practices in Michigan. Thirty-eight practices with 71 distinct practice sites collaborate to advance the quality and value of cancer care throughout the state. MOQC is sponsored by the Value Partnerships program of Blue Cross Blue Shield of Michigan (BCBSM) but collects data on patient care regardless of their insurance status or type. Across the practices in the collaborative, there are 14 different electronic health records. The state is divided into 6 geographic regions for twice-yearly regional meetings. Performance data are also shared at 2 collaborative-wide meetings each year. At least 1 physician and at least 1 other practice member, such as an administrator or other practice leader, attend these regional meetings and at least 1 of 2 collaborative-wide meetings each year. MOQC practices that meet specific performance targets as well as the attendance requirement are eligible for reimbursement in accordance with the BCBSM value-based reimbursement fee schedule, which sets reimbursement at greater than 100% of the standard fee schedule, ranging from 2% to 7% based on reaching target performance on several measures. Targets for each measure are chosen annually to exceed performance during the preceding year, encouraging continuous improvement. Measures and targets are chosen by a multidisciplinary committee of practice members and members of the Patient and Caregiver Oncology Council.

### Measures

MOQC began measuring guideline-concordant HEC prophylaxis with a 4-drug regimen of olanzapine added to a corticosteroid, a 5HT3RA, and an NK1RA with the first cycle of chemotherapy in 2019. This measure was developed by the ASCO Quality Oncology Practice Initiative (QOPI). QOPI is a voluntary data abstraction registry through ASCO that helps oncology practices assess the quality of the care they provide to patients against evidence- and consensus-based standards.^16^

The numerator for this measure includes patients who received olanzapine as part of their antiemetic regimen with the first cycle of treatment with an HEC regimen. The denominator is all patients in the practice receiving their first cycle of HEC. Patients in an antiemetic clinical trial were excluded. Each patient contributed only 1 data point; prescribing of olanzapine in subsequent cycles is not measured.

### Data Collection

Data were manually abstracted from health records into the QOPI database throughout the year by trained abstractors through December 2022. The same abstractors and abstraction schedule was maintained upon the change to a new database in January 2023. Data collection is done in 2 prespecified 6-month rounds per year so that comparisons can be made round-over-round and year-over-year. With the move to the MOQCLink database in January of 2023, data could be analyzed and presented at any time, such as at regional meetings. Case identification dates (for diagnosis and office visits) are set by QOPI and were preserved upon the transition to MOQCLink. Data are abstracted from the records of patients with a cancer diagnosis at their first office visit that occurred no more than 18 months before each collection round. The 18-month window ensures that all eligible patients seen in the practice are included in the database. Data are entered only once for each patient, and each patient contributed only 1 data point. Audits are performed to ensure data accuracy and completeness.

### Interventions

Implementation in each practice was led by the clinical champion with support from the practice manager, and if applicable, the practice pharmacist. These practice members led the work to identify the specific challenges in their practice and use their experience to make practice-wide changes to, for example, antiemetic order sets. Each round of data allows practices to conduct quality improvement cycles at their own sites. MOQC does not specify how practices are to enact quality improvement given the unique characteristics of each practice and the belief that practices are best positioned to identify barriers and countermeasures in their practices.^17^

The interventions deployed were selected based on established quality improvement methods and on needs identified by MOQC practices as well as established ways of incentivizing improvement across all the value partnership collaboratives sponsored by BCBSM. The interventions were the following: (1) performance audit and feedback^18,19^ with peer comparison at collaborative meetings and upon request by practices; (2) prescriber education by experts at collaborative-wide meetings; (3) learning collaboratives, in which practices shared the barriers they faced to prescribing and the ways in which they addressed those barriers; (4) the development of patient-facing information about olanzapine; and (5) value-based reimbursement for all practices in the regions that met the prescribing target. These interventions have all been shown to improve quality within our consortium and in published literature on changing prescriber behavior. Each of these is described in more detail in the following paragraphs.

Audit and feedback:^18,19^ practice performance data are shared at 4 meetings per year. Practice-, region-, and collaborative-level data are shown alongside one another so that practices can compare their performance with others. Additional support provided to practices as of 2023 includes providing access to their data via a live dashboard, allowing practices to see their performance data in real time, and breaking down data by practice location and physician. This allows practices to target gaps in care and act quickly to change prescribing behavior. Learning collaboratives:^20,21^ during regional meetings, practice members from both high and low performing practices shared barriers to olanzapine prescribing and strategies for improving performance.

Prescriber education by experts:^20,21,22^ at 2 collaborative-wide meetings (June 2019 and January 2022), MOQC invited 2 experts to give formal presentations about the mechanism of chemotherapy-induced nausea and vomiting, the evidence supporting the antiemetic prescribing guidelines, the indications for and dose range of olanzapine, and the cost of the drug. Patient-facing information about olanzapine: the MOQC Coordinating Center created a 1-page patient facing resource in collaboration with oncology pharmacists, patients, and caregivers in response to reports from clinicians that patients often decline the use of olanzapine given its labeling as an antipsychotic and concerns about cost.

Value-based reimbursement:^23,24^ prescribing of olanzapine in patients receiving HEC was selected as 1 component of a set of value-based reimbursements in 2019. The performance target was initially set at 25% based on the performance at that time. MOQC uses an adaptive approach to setting targets that exceed current performance but are attainable. The target in 2023 was increased to 30% in 2023 and then again to 55% in 2024.

### Statistical Analysis

Measure calculation was performed by QOPI between 2019 and 2022. Beginning in 2023, the internal MOQC data analytics team performed the same calculation based on the numerator and denominator criteria described in the Measures section.

Data were aggregated by practice and for the entire collaborative. Continuity-adjusted χ^2^ tests were performed to calculate the statistical differences between performance for each round compared with the preceding round and for the trend over time. A 2-sided *P* of less than .05 was considered significant. All analyses were performed using R version 4.4.0 (R Project for Statistical Computing.

## Results

The patient sample included 8662 patients treated in 38 practices at 71 sites by 352 medical oncologists. The mean number of patients per clinician per round was 3.2 (median 2, range 1-23). Individual patient-level demographic data are not available from the QOPI database. Characteristics of patients from MOQCLink (years 2021 through 2024) are shown in Table 1 (median [IQR] age, 62 [52-69] years; 4434 female patients [65.5%]; 814 Black or African American [12.0%], 121 Hispanic or Latino [1.8%], and 5385 White [79.7%]).

**Table 1.** Patient Characteristics in Michigan Oncology Quality Consortium Link, 2021-2024

Characteristic	Patients, No. (%)
Sex	
Male	2338 (34.5)
Female	4434 (65.5)
Race	
American Indian or Alaskan Native	34 (0.5)
Asian	94 (1.4)
Black or African American	814 (12.0)
Native Hawaiian or Pacific Islander	5 (0.17)
White	5385 (79.7)
Not reported	172 (2.5)
Other^a^	137 (2.0)
≥2	36 (0.5)
Unknown	83 (1.2)
Ethnicity	
Not Hispanic or Latino	5395 (79.86)
Hispanic or Latino	121 (1.8)
Not reported or unknown	480 (7.1)
Unknown	747 (11.0)
Other	21 (0.3)
Age, y	
Mean	60.15
Median (IQR) [range]	62 (52-69) [18-98]

^a^ Patients chose “other” and provided no further detail.

**Figure 1.**
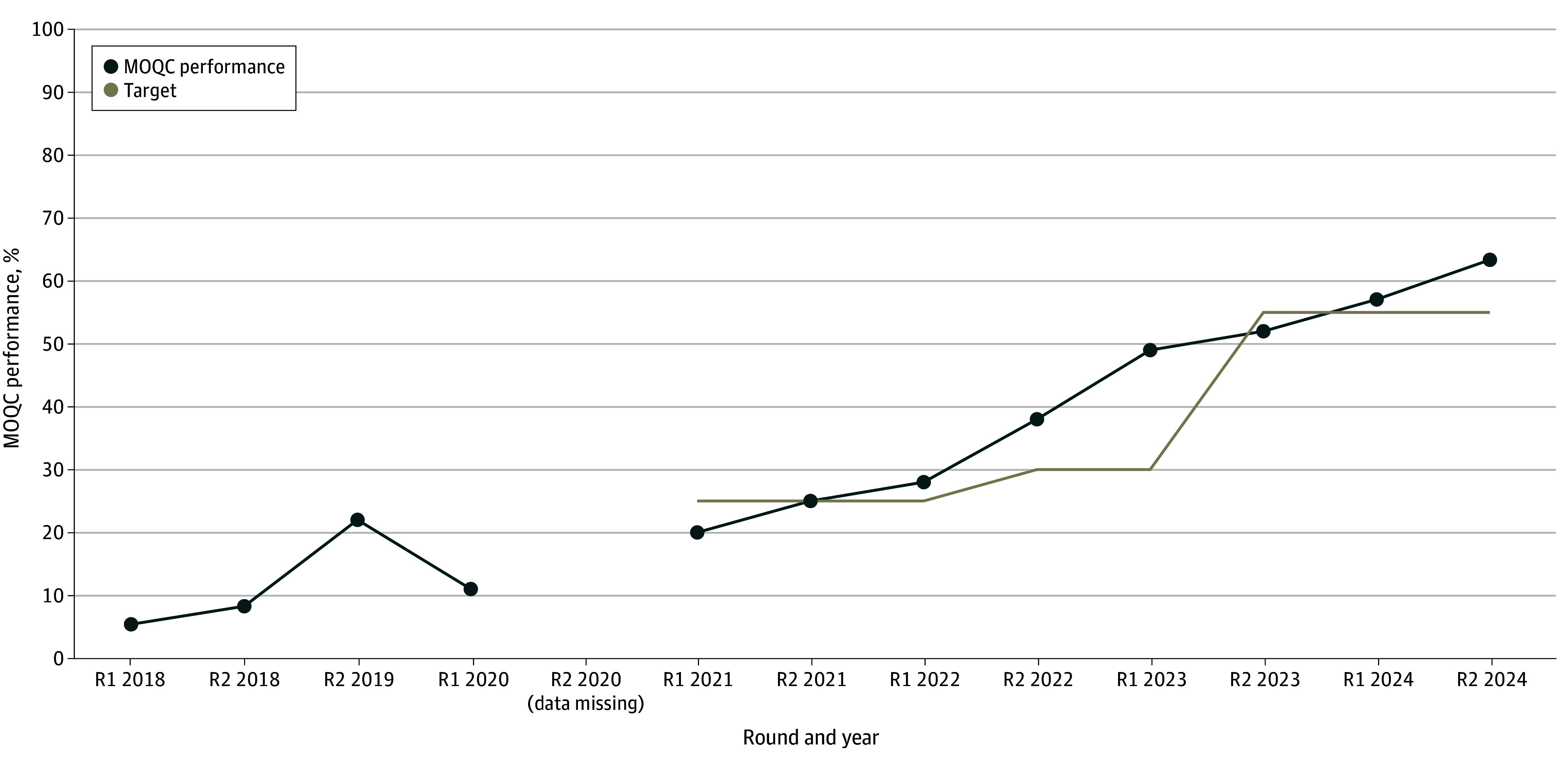
The Proportion of Patients Receiving Olanzapine as Part of a 4-Drug Antiemetic Regimen for the First Cycle of Highly Emetogenic Chemotherapy Over Time Data are collected in 2 rounds per year as designated by R1 and R2. Data are not available for Round 2 of 2020 due to an error in the Quality Oncology Practice Initiative database. MOQC indicates Michigan Oncology Quality Consortium; R, round.

The interventions made within practices to increase the use of olanzapine included sharing performance data in the practice compared with others in the collaborative and region, education about the antiemetic guidelines, changes to HEC order sets to include olanzapine, and inclusion of patient-facing information about olanzapine in chemotherapy teaching sessions. In round 1 of 2019, 63 of 880 patients treated with HEC (7.2%) received olanzapine as part of a 4-drug regimen. This increased to 422 of 666 patients (63.4%) in round 2 of 2024 (χ^2^_1_ = 553.61; *P* < .001), compared with round 1 of 2019, exceeding the target of 55%. Figure 1 shows the change in performance over time. Data for round 2 of 2020 were missing due to a vendor error. The mean, ranges, IQRs, and standard deviation for each round are shown in Table 2.

**Table 2.** The Percentage of Patients Receiving Olanzapine as Part of a 4-Drug Antiemetic Regimen for Highly Emetogenic Chemotherapy Over Time

Year (round)^a^	Patients, No./total No. (%)	Patients, Median (Q1-Q3) [range], %	*P* value^b^
2019 (1)	63/880 (7)	0 (0-0) [0-22]	NA
2019 (2)	123/692 (13)	0 (0-10) [0-43]	<.001
2020 (1)	162/977 (17)	0 (0-9) [0-75]	.01
2020 (2)^c^	NA	NA	NA
2021 (1)	218/1041 (21)	0 (0-9) [0-91]	.01
2021 (2)	235/873 (27)	6 (0-42) [0-100]	.00
2022 (1)	280/960 (29)	11 (0-40) [0-100]	.14
2022 (2)	359/948 (38)	0 (0-25) [0-93]	<.001
2023 (1)	331/657 (50)	45 (12-68) [0-100]	<.001
2023 (2)	376/719 (52)	43 (24-71) [0-100]	.20
2024 (1)	395/695 (57)	61 (17-75) [0-100]	.21
2024 (2)	422/666 (63)	70 (44-98) [0-100]	.02

Abbreviations: NA, not available; Q, quartile.

^a^ Data are collected in 2 rounds per year as indicated by 1 and 2 in the table.

^b^ *P* values show significance round-over-round.

^c^ Data not available for 2020 (round 2) due to a vendor error.

**Figure 2.**
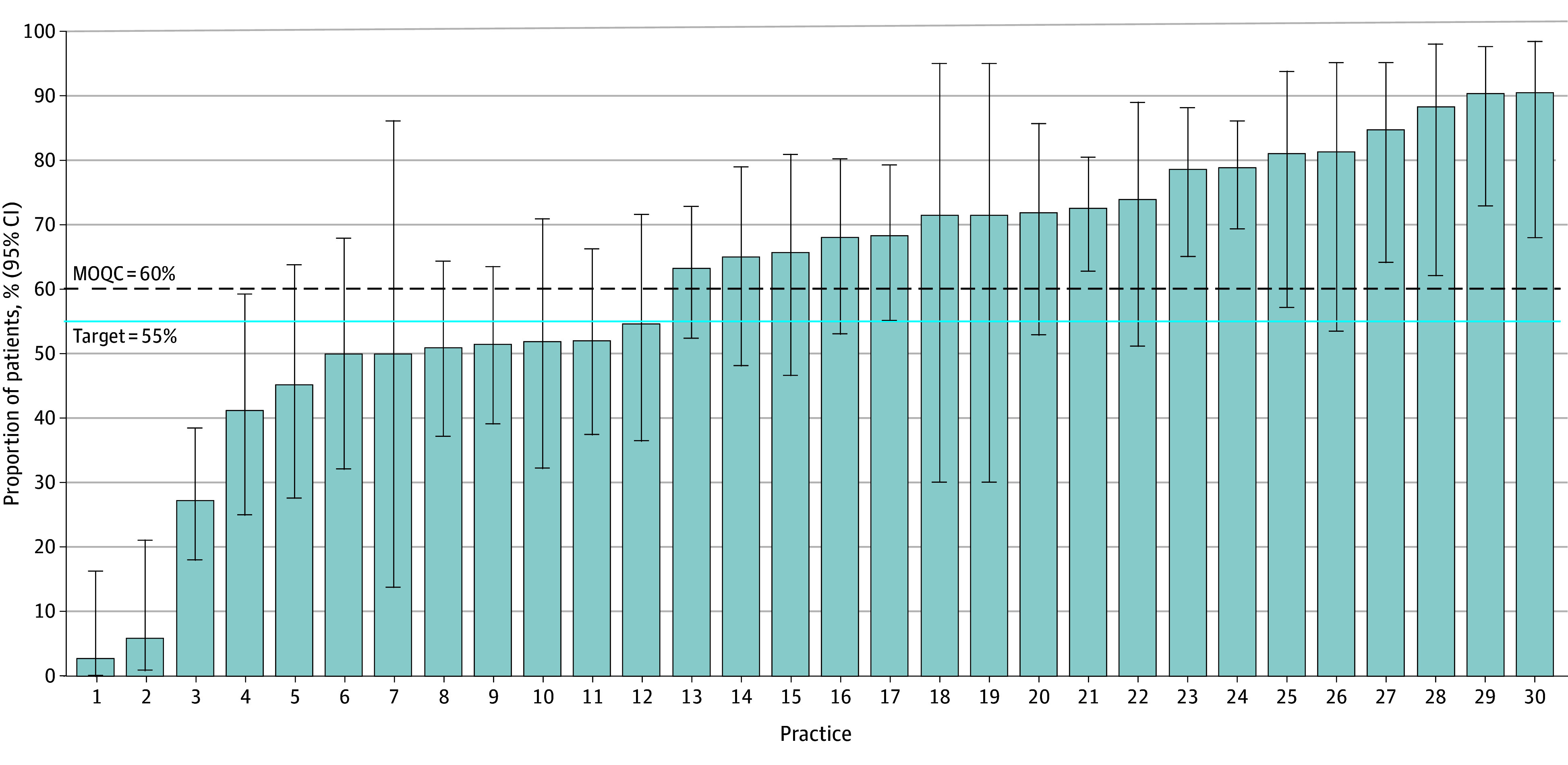
Proportion of Patients Receiving Highly Emetogenic Chemotherapy Who Were Prescribed Olanzapine With the First Cycle by Practice, January 1 Through December 31, 2024 (n = 1254) Only practices who contributed 5 or more patients are included in this figure. The vertical bars indicate the percentage of patients in the MOQC database across all practices who were prescribed olanzapine, and error bars represent 95% CIs. MOQC indicates Michigan Oncology Quality Consortium.

Variation in performance across practices persisted throughout the initiative, including during the most recent year of data as shown in Figure 2. Only those practices that submitted more than 5 patient records are included in the figure. In the most recent 12 months, the proportion of patients in a practice receiving olanzapine varied by practice from 0% to 100%. Twenty-one practices (66% of practices that submitted more than 5 patient records) reached or exceeded the target performance. Six practices were prescribing olanzapine to at least 75% of their patients, and 6 were prescribing olanzapine to fewer than 10% of their patients receiving HEC. Practice members of those practices not prescribing olanzapine have cited patient reluctance to take a drug labeled as an antipsychotic and the belief that olanzapine is not necessary in cycle 1 of HEC.

## Discussion

In summary, practices in this statewide collaborative increased first-cycle guideline-concordant olanzapine prescribing in patients receiving HEC from less than 10% to over 50% over a 5-year period using performance audit and feedback with peer-comparison, expert education for prescribers, learning collaboratives of practice measures, patient education, and value-based reimbursement.

Olanzapine prescribing in people receiving HEC has been persistently low across the US. In a sample of over 60 000 patients in a commercial database treated through 2021, fewer than 14% of patients treated with HEC were prescribed olanzapine.^13^ Previous work has shown that it takes approximately 15 years for cancer control guidelines or the development of evidence-based practices to disseminate into clinical care,^25^ so the delay in uptake is not surprising.

Reasons for underuse of olanzapine likely include the labeling of olanzapine as an atypical antipsychotic and the resultant hesitation on the part of patients and prescribers reported by our practices, underreporting of nausea and vomiting by patients,^1^ underestimation of the extent and impact of CINV by prescribers,^1^ and lack of awareness of or agreement with the guidelines.^26^

We believe that the success of our quality improvement work resides primarily in our culture of quality improvement that draws from the expertise and experiences of our practice members.^17^ The specific approaches that we use, including the regular sharing of performance data and peer comparisons, encourages practice members to learn from one another and to adapt successful strategies in their own practices. Our regional meetings serve as learning collaboratives of prescribers and pharmacists at diverse practices and have been effective in expanding both knowledge and the willingness to change practice. Most of our practices have also been able to overcome objections to the use of olanzapine, including concerns about the stigma associated with the use of an atypical antipsychotic and concerns about the sedating effects of olanzapine. Value-based reimbursement increases the salience of specific measures and supports practice change. Additionally, the MOQC committee that selects measures has been persistent about encouraging guideline concordant prescribing of antiemetics.

Most published work has demonstrated relatively small effects of both audit and feedback^18^ and clinician education by experts. Peer comparisons have similarly shown small changes in prescribing behavior.^19^ Value-based payments have been shown to improve performance by approximately 5%. It is thus likely that combined strategies, as we have implemented, are needed to support sustained quality improvement.

Ongoing work includes continued information support and coaching in practices with low olanzapine uptake and providing targeted assistance and resources to ensure adoption of effective CINV management strategies across all member practices. We continue to raise the target for performance each year and hope to achieve a 90% prescribing rate over the next few years. We are also investigating the relationship between increased olanzapine prescribing and health care utilization, including hospitalization associated with HEC.

### Limitations

The limitations of our work include the small number of patients per prescriber, which does not allow us to evaluate and understand changes in prescribing over time at the physician level. We also do not specify which methods are to be used by the practices to improve quality, which may limit the reproducibility of our work. Additionally, we lack information regarding the prevalence of CINV in our patient sample and thus cannot draw conclusions about the impact of our quality improvement work on patients directly. Despite this limitation, the impact of CINV on patient quality of life and on costs of care has led us to surmise that improving the uptake of olanzapine is likely to be a low-cost intervention to improve quality of life. One hospital admission for CINV costs a mean of $15 000^27^ compared with the cost of olanzapine (less than $20 for thirty 5-mg tablets). Widespread use of olanzapine, as seen in MOQC practices, is thus likely to have an impact on the costs of care associated with chemotherapy.

## Conclusions

In this quality improvement study, MOQC demonstrated significantly increased olanzapine prescribing in patients receiving HEC to an average well above that seen nationally in diverse practices across multiple practices and health systems employing strategies that are adaptable to local practice environments and resources. Our approaches to improving quality can be used by others to increase guideline-concordant prescribing of olanzapine across diverse settings, which in turn is likely to improve patient quality of life and to reduce health care costs.

## Notes

Supplement.
Data Sharing Statement
