# Supplementary material for: Increasing Olanzapine Prescribing for Patients Undergoing Highly Emetogenic Chemotherapy
Source: JAMA Netw Open. 2025 May 21;8(5):e2510392. doi: 10.1001/jamanetworkopen.2025.10392 (PMC12096248; doi:10.1001/jamanetworkopen.2025.10392)
Supplement: Supplement. — Data Sharing Statement [file jamanetwopen-e2510392-s001.pdf]

Data Sharing Statement

Bowen. Increasing Olanzapine Prescribing for Highly Emetogenic Chemotherapy in a Statewide Collaborative. *JAMA Netw Open*. Published May 21, 2025. doi:10.1001/jamanetworkopen.2025.10392

Data
Data available: No
